# Supplementary material for: Occurrence of Nine Pyrrolizidine Alkaloids in Senecio vulgaris L. Depending on Developmental Stage and Season
Source: Plants (Basel). 2019 Mar 5;8(3):54. doi: 10.3390/plants8030054 (PMC6473320; doi:10.3390/plants8030054)
Supplement: Supplementary file 1 [file plants-08-00054-s001.pdf]

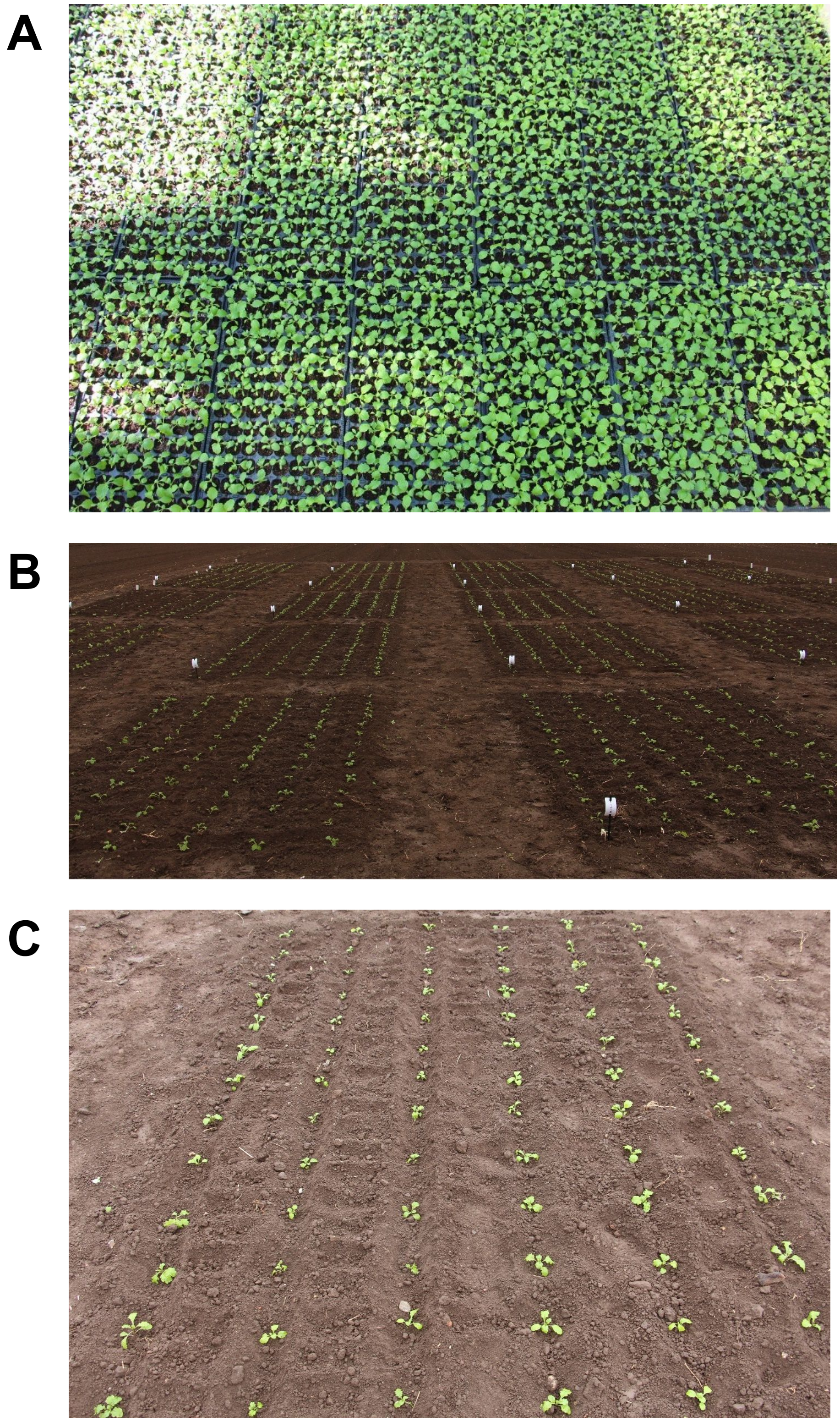

Supplemental Figure S1. A: Plants of *Senecio vulgaris* L. during cultivation in greenhouse for experimental part "autumn" in TEKU®-palettes (Pöppelmann, Lohne, Germany) for 104 plants per pallet, with a size of 55 x 28 x 3.8 cm and a volume of 21 mL per pot (16 August, 2016); B: Plants of *Senecio vulgaris* L. totally planted out for experimental part "spring": 25 field plots scaling 5.54 m^2 each (30 March, 2016); C: Plants of *Senecio vulgaris* L. planted out for experimental part "spring", one plot with a scale of 2.8 × 1.98 m (30 March, 2016).

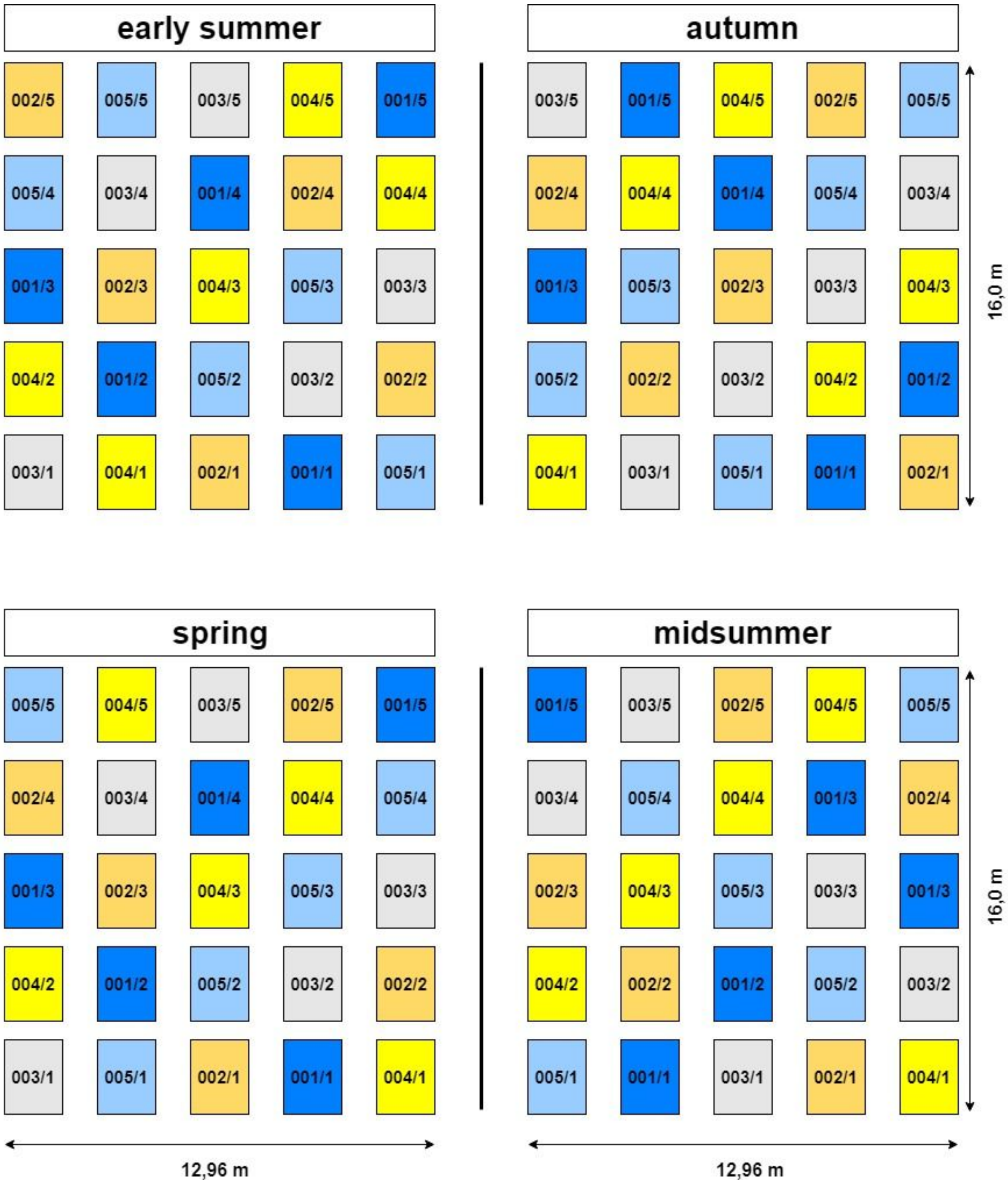

Supplemental Figure S2. Schematic representation of the complete experimental design as Latin squares for the effect stage for each sub trial, in a general linear model with: $y_{ijk}=\mu+\alpha_i+c_j+r_k+e_{ijk}$ (μ=mean; α=stage; c=column, r=row). First number represents the developmental stage, second number the replicate number. For example: 004/3 represents the 3rd replicate to investigate developmental stage 4 of the distinct season.

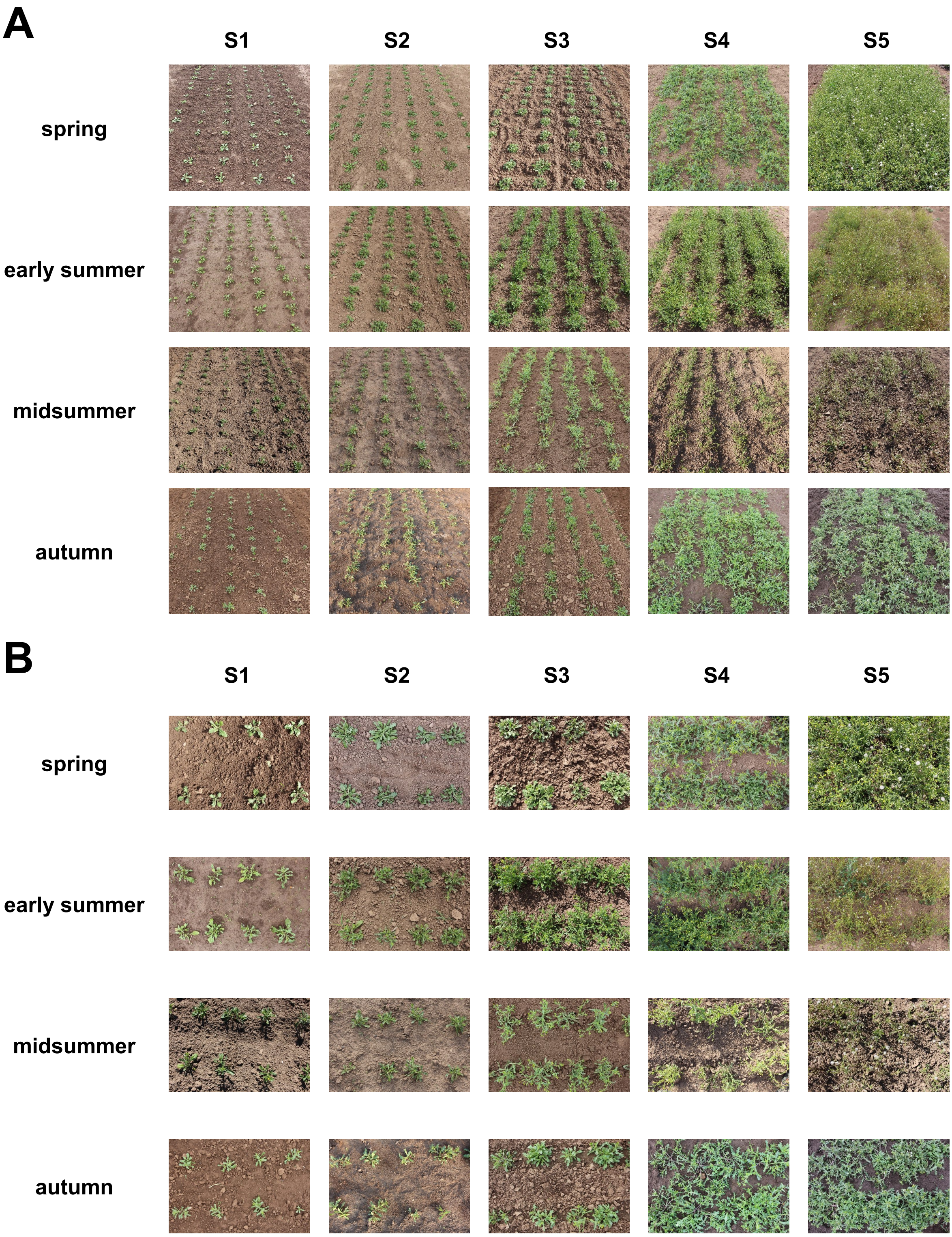

Supplemental Figure S3. A: **Field plots of** *Senecio vulgaris* L. before sampling, representative pictures; B: **Plants of** *Senecio vulgaris* L. before sampling, representative pictures.

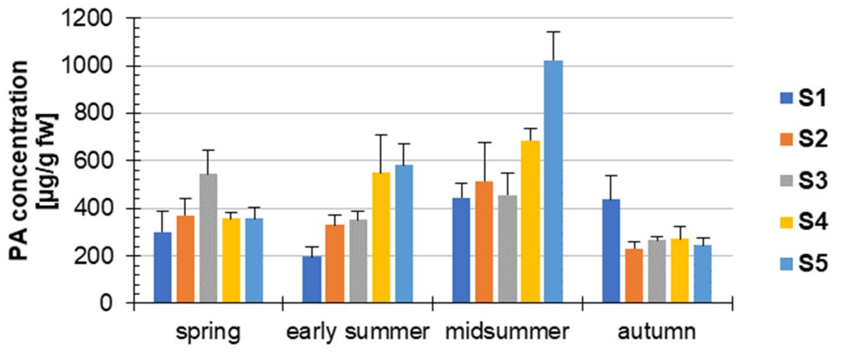

Supplemental Figure S4. Total PA concentration (µg/g) of nine different PAs in *Senecio vulgaris* L. plants (fresh weight) depending on the developmental stage (S1 to S5) and season. Results are means +/- SD of five different determinations (distinct sub trials, see Supplemental Figure S2 and S3).

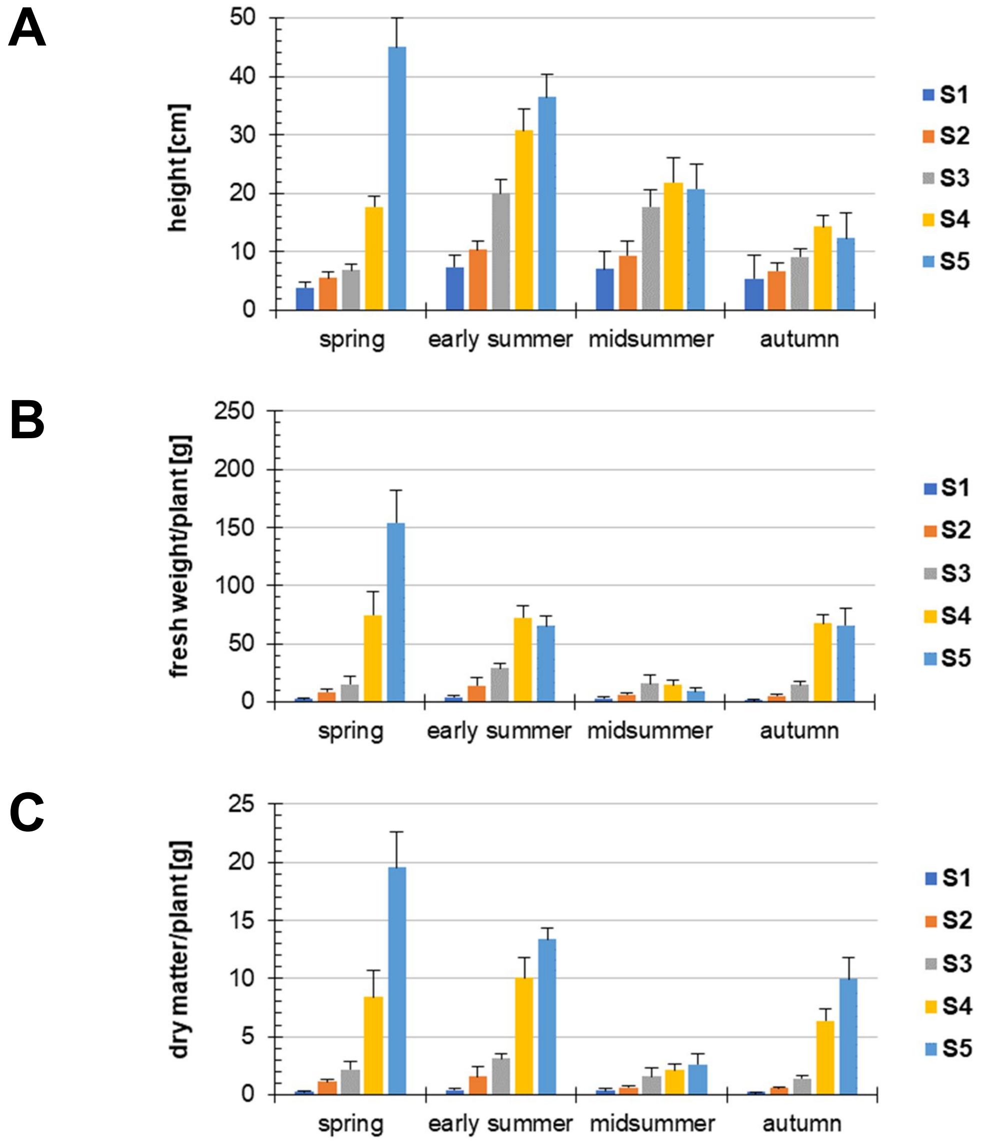

Supplemental Figure S5. A: Average height (cm) of *Senecio vulgaris* L. plants depending on the developmental stage (S1 to S5) and season. B: Average fresh weight (g) of *Senecio vulgaris* L. plants depending on the developmental stage (S1 to S5) and season. C: Average dry matter (g) of *Senecio vulgaris* L. plants depending on the developmental stage (S1 to S5) and season. Results are means +/- SD of five different determinations (distinct sub trials, see Supplemental Figure S2 and S3).

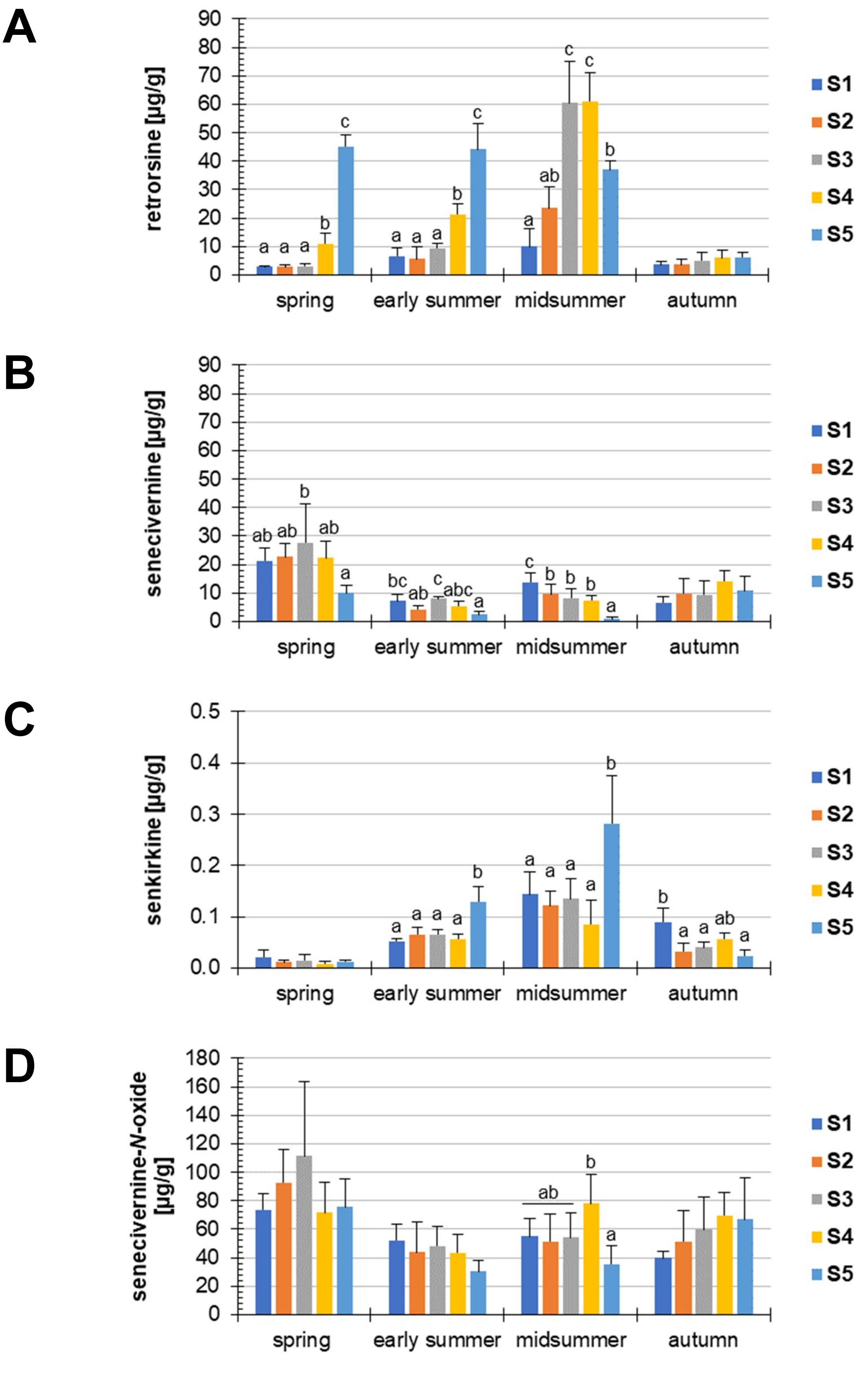

Supplemental Figure S6. PA concentration (µg/g) of A: retrorsine; B: senecivernine; C: senkirkine; D: senecivernine-*N*-oxide in *Senecio vulgaris* L. plants (dry mass) depending on the developmental stage (S1 to S5) and season. Results are means +/- SD of five different determinations (distinct areas, see Supplemental Figure S2). Different letters identify significant differences ($p < 0.05$) between developmental stages (ANOVA, Tukey's honest significance difference).
